# Development of an Algorithm to Assist in the Diagnosis of Combined Retinal Vein Occlusion and Glaucoma

**DOI:** 10.3390/jcm14238547

**Published:** 2025-12-02

**Authors:** Hiroshi Kasai, Kazuyoshi Kitamura, Yuka Hasebe, Junya Mizutani, Kengo Utsunomiya, Shiori Sato, Kohei Murao, Yoichiro Ninomiya, Kensaku Mori, Kazuhide Kawase, Masaki Tanito, Toru Nakazawa, Atsuya Miki, Kazuhiko Mori, Takeshi Yoshitomi, Kenji Kashiwagi

**Affiliations:** 1 Department of Ophthalmology, Faculty of Medicine, University of Yamanashi, Yamanashi 409-3898, Japan; 2 Research Center for Medical Big Data (RCMB), National Institute of Informatics, Tokyo 101-8430, Japan; 3 Yasuma Eye Clinic, Nagoya 460-0011, Japan; 4 Department of Ophthalmology Protective Care for Sensory Disorders, Nagoya University Graduate School of Medicine, Nagoya 466-8550, Japan; 5 Department of Ophthalmology, Shimane University Faculty of Medicine, Izumo 693-0021, Japan; mtanito@med.shimane-u.ac.jp; 6 Department of Ophthalmology, Tohoku University Graduate School of Medicine, Sendai 980-8575, Japan; 7 Department of Myopia Control Research, Aichi Medical University, Nagaokakyo-city 617-0826, Japan; 8 Baptist Eye Institute, Kyoko 606-8287, Japan; kmori@koto.kpu-m.ac.jp; 9 Department of Ophthalmology, Kyoto Prefectural University of Medicine, Kyoto 602-8566, Japan; 10 Department of Orthoptics, Fukuoka International University of Health and Welfare, Fukuoka 814-0001, Japan

**Keywords:** artificial intelligence, glaucoma, retinal vein occlusion (RVO)

## Abstract

**Objectives:** To develop an algorithm to assist in the diagnosis of glaucoma with concomitant retinal vein occlusion (RVO) and to compare its diagnostic accuracy with that of ophthalmology residents and specialists. **Methods:** Fundus photographs of eyes with RVO and those with both RVO and glaucoma were obtained from patients who visited the University of Yamanashi Hospital. All images were preprocessed through normalization and resized to 512 × 512 pixels to ensure uniformity before model training. The diagnostic accuracy of two algorithms—the Comprehensive Fundus Disease Diagnostic Artificial Intelligence Algorithm (CD-AI) and the Glaucoma Concomitant RVO Artificial Intelligence Algorithm (RVO-GLA AI)—was evaluated. CD-AI is a clinical decision support algorithm originally developed to detect eleven common fundus diseases, including glaucoma and RVO. RVO-GLA AI is a fine-tuned version of CD-AI that is specifically adapted to detect glaucoma with or without RVO. Fine-tuning was performed using 1234 images of glaucoma, 1233 images of nonglaucomatous conditions, including RVO, and 15 images of cases with both glaucoma and RVO. The number of comorbid cases was determined empirically by gradually adding glaucomatous eyes with concomitant RVO to the training set, and 15 images provided the best balance between sensitivity and specificity. Because the available number of such cases was limited, this small sample size may have influenced the stability of the performance estimates. For the final evaluation, both algorithms and all ophthalmologists assessed the same independent test dataset comprising 66 fundus images (16 eyes with glaucoma and RVO and 50 eyes with RVO alone). The diagnostic performance of both algorithms was compared with that of three first-year ophthalmology residents and three board-certified ophthalmologists. **Results:** CD-AI demonstrated high diagnostic accuracy (92.5%) in eyes with glaucoma alone. However, its sensitivity and specificity decreased to 0.375 and 1.0, respectively, in patients with concomitant RVO. In contrast, the RVO-GLA AI achieved an area under the curve (AUC) of 0.875, with a sensitivity of 0.87 and a specificity of 0.71. Across all the ophthalmologists, the average sensitivity was 0.63, and the specificity was 0.87. Specialists achieved a sensitivity of 0.80 and a specificity of 0.89, while residents had a sensitivity of 0.46 and a specificity of 0.85. **Conclusions:** An AI-based clinical decision support system specifically designed for glaucoma detection significantly improved diagnostic performance in eyes with combined RVO and glaucoma, achieving an accuracy comparable to that of ophthalmologists, even with a limited number of training cases.

## 1. Introduction

Glaucoma is among the leading causes of blindness worldwide [1,2]. Since the visual dysfunction associated with glaucoma is progressive and irreversible, early detection is critical for preventing blindness. However, early detection remains challenging because the disease is largely asymptomatic in its early stages. Clinical decision support systems based on artificial intelligence (AI) have garnered significant attention in recent years. Numerous studies have demonstrated the potential of deep learning algorithms to detect glaucoma with diagnostic accuracies comparable to those of ophthalmology specialists [3,4,5,6,7,8,9,10]. In a study by Li et al., who developed a glaucoma detection algorithm using 48,116 fundus photographs, the area under the curve (AUC) for glaucoma diagnosis was reported to be 0.986, with a sensitivity of 95.6% and a specificity of 92.0%, indicating excellent diagnostic performance [4]. In our investigation using color fundus photographs of Japanese patients, we also evaluated the diagnostic accuracy of three deep convolutional neural network (DCNN) models for glaucoma. All the models achieved AUCs exceeding 0.9, demonstrating favorable performance [3]. These findings suggest that artificial intelligence models may represent promising candidates for glaucoma screening.

Despite these advancements, most prior studies have focused on diagnosing single diseases using AI-based algorithms. In real-world clinical practice, patients often present with multiple coexisting pathologies. Li et al. further reported that a substantial proportion of false-negative cases involved glaucomatous optic neuropathy accompanied by additional ocular findings or comorbid conditions unrelated to glaucoma [4]. The utility of AI-based algorithms for detecting glaucoma in the presence of cooccurring pathologies has not been sufficiently investigated. Accurate diagnosis in such complex cases is typically more challenging for clinicians than for cases involving a single disease. Therefore, evaluating the performance of AI-based clinical decision support systems in these scenarios is essential for assessing their practical applicability in real-world settings.

Previous studies investigating the association between glaucoma and retinal vein occlusion (RVO) have yielded inconsistent results, with some reporting a positive association [11] and others finding no significant relationship [12]. Yin et al., however, conducted a meta-analysis on this topic and reported that, across prior studies, glaucoma was associated with an odds ratio (OR) of 4.01 (95% confidence interval [CI]: 3.28–4.91) as a risk factor for RVO [13]. Rogers et al. reported that RVO is a common ophthalmic condition with a prevalence of approximately 2% [14]. In clinical practice, it is not uncommon to encounter patients who present with both glaucoma and RVO. In such cases, flame-shaped retinal hemorrhages, venous dilation, macular edema, and disc swelling or pallor can obscure glaucomatous cupping and retinal nerve fiber layer defects, making both clinical judgment and automated classification more difficult. Moreover, both glaucoma and RVO are thought to involve vascular dysregulation and endothelial dysfunction, which may partially explain their co-occurrence.

While many previous studies have focused on distinguishing eyes with a target fundus disease from normal eyes without any fundus pathology, few have explored the differentiation of glaucoma from other fundus diseases, whether as single or multiple conditions. The co-occurrence of glaucoma and RVO, although not rare, presents challenges in the collection of a sufficiently large dataset for algorithm development. This limitation poses a significant barrier to creating robust AI-based diagnostic tools. The development of algorithms capable of performing effectively with relatively small sample sizes is necessary to overcome this issue.

In this study, we aimed to develop an AI-based algorithm for diagnosing glaucoma in patients with concomitant RVO. We also compared the diagnostic accuracy of our algorithm to those of previously developed algorithms and ophthalmologists to evaluate its performance under real-world conditions. We hypothesized that a fine-tuned AI model incorporating co-pathological data from eyes with glaucoma and RVO could enhance diagnostic accuracy compared with standard single-disease models.

## 2. Materials and Methods

This study was approved by the Ethics Committee of the University of Yamanashi School of Medicine (approval code: 2635; approval date: 6 March 2023). All procedures were conducted in accordance with the principles outlined in the Declaration of Helsinki. All the images used in this study have been anonymized, and the ethics committee has waived the requirement for informed consent. Investigators accessed the data for research purposes on 5 November 2023.

### 2.1. Development of a Comprehensive Fundus Disease Diagnostic Artificial Intelligence Algorithm (CD-AI)

The comprehensive diagnostic AI (CD-AI) algorithm was developed collaboratively by the Japanese Society of Artificial Intelligence in Ophthalmology and the National Institute of Informatics. A total of 13,452 fundus photographs, including images from patients with one of eleven fundus diseases (glaucoma, age-related macular degeneration, central serous chorioretinopathy, retinal vein occlusion, macular hole, epiretinal membrane, diabetic retinopathy, myopic chorioretinopathy, macular edema, retinitis pigmentosa, and non-glaucoma optic atrophy) and normal controls, were collected from 14 Japanese university hospitals [15]. The diagnoses for each image were confirmed by at least two ophthalmology experts, and all the images were taken using a fundus camera at a 45° angle.

The base algorithm for CD-AI was VGG19, trained on 8000 fundus images from this multi-center dataset, as previously described [15]. The algorithm assigns “possibility” scores for each disease, ensuring that the probabilities for all diseases sum to 1.0. Detailed diagnostic accuracy metrics for CD-AI were obtained from internal unpublished data. In brief, the diagnostic performance of this support algorithm for glaucoma alone or RVO alone was 0.92 classification accuracy (unpublished data). This study specifically evaluated the diagnostic accuracy of CD-AI for detecting glaucoma in eyes with coexisting glaucoma and RVO.

### 2.2. Enrollment Criteria for Fundus Images

All images for the development and evaluation of the RVO-GLA AI algorithm were obtained at the University of Yamanashi Hospital. Fundus images were captured using a Topcon TRC-NW400 fundus camera (Topcon, Tokyo, Japan) (45° field, JPEG format, 2048 × 1536 pixels). RVO diagnosis was based on clinical findings such as flame-shaped retinal hemorrhages, venous occlusion at arteriovenous crossings, and white line veins, confirmed by fluorescein angiography or optical coherence tomography (OCT) angiography. Patients with diabetes mellitus (DM) or typical diabetic retinopathy in the unaffected eyes were excluded. Macular RVO patients were further screened with OCT and OCT angiography to exclude age-related macular degeneration.

Patients with concomitant glaucoma and RVO had either been diagnosed and treated for glaucoma prior to developing RVO or were diagnosed with glaucoma during follow-up for RVO. Glaucoma diagnosis required confirmation of glaucomatous optic neuropathy (GON) and nerve fiber layer thinning via OCT, along with corresponding visual field defects confirmed by static perimetry. The final diagnoses were made by glaucoma specialists at the University of Yamanashi Glaucoma Outpatient Clinic.

### 2.3. Development of an Algorithm for Diagnosing Glaucoma in Eyes with Concomitant RVO (RVO-GLA AI)

All images for the development of the RVO-GLA AI algorithm were obtained at the University of Yamanashi Hospital.

The inclusion and exclusion criteria for RVO and glaucoma were identical to those described in the “Enrollment criteria for fundus images” section. This study included 67 images of eyes with glaucoma and RVO and 412 images of eyes with RVO alone.

### 2.4. Training and Validation of the RVO-GLA AI

All images were preprocessed through normalization and resized to 512 × 512 pixels to ensure uniformity before model training. As part of the tuning process in the development of the RVO-GLA AI, the base model was changed from VGG19 to EfficientNetB4. From the 8000 images used for the development of CD-AI, we extracted 1234 glaucomatous images and 1233 non-glaucomatous images randomly selected from 6766 non-glaucomatous cases. The non-glaucomatous images were evenly distributed across all disease classes. A total of 2467 images were used to train the glaucoma diagnostic model. The RVO-GLA AI was implemented in TensorFlow and trained by fine-tuning the EfficientNetB4-based glaucoma classifier. Training was performed using the Adam optimizer with a learning rate of 1 × 10^−4^, a batch size of 16, and 10 epochs. During training, data augmentation consisted of horizontal and vertical flips and random width and height shifts (each up to 0.1 of the image dimension); no rotation was applied, and no brightness or color adjustments were used.

To further refine this glaucoma diagnostic model for the RVO-GLA AI, glaucomatous images with concomitant retinal vein occlusion (RVO) were gradually added to the training dataset as positive (glaucoma) cases. For evaluation, an independent test dataset, which had not been used during the tuning process, was employed. This dataset consisted of 412 RVO-only images and 67 glaucomatous images with concurrent RVO. Thus, the training set (2467 images) and the independent test set (489 images) were completely non-overlapping. Diagnostic performance was assessed under the following conditions, with the resulting sensitivity and specificity reported below: (1) Training without any comorbid cases yielded a sensitivity of 0.299 and a specificity of 0.871. (2) Training with 10 comorbid cases yielded a sensitivity of 0.881 and a specificity of 0.447. (3) Training with 15 comorbid cases yielded a sensitivity of 0.790 and a specificity of 0.755.

On the basis of these findings, the model trained with 15 comorbid cases (Model 3) was adopted as the final version of the RVO-GLA AI.

For the final evaluation, 50 RVO-only images and 16 glaucomatous images with concurrent RVO, which had not been used for training, were assessed by the RVO-GLA AI, board-certified ophthalmologists of the Japanese Ophthalmological Society, and ophthalmology residents.

Diagnostic performance metrics, including sensitivity and specificity, were calculated for the RVO-GLA AI and for each ophthalmologist group. Sensitivity and specificity were reported together with their 95% confidence intervals, which were derived from the binomial distribution using the Wilson score method.

To compare paired diagnostic outcomes between the RVO-GLA AI and ophthalmologists, we applied McNemar’s test to 2 × 2 contingency tables of correct versus incorrect classifications. A *p*-value < 0.05 was considered statistically significant.

## 3. Results

### 3.1. Diagnostic Accuracy of the CD-AI for Glaucoma in Eyes with or Without RVO

From the collected fundus image dataset, 40 glaucoma cases that were not used in AI development were randomly selected to evaluate the diagnostic accuracy. A diagnosis was considered correct when glaucoma was the top-ranked output and incorrect when glaucoma was ranked second or lower.

CD-AI demonstrated a high accuracy of 92.5% in 40 eyes with glaucoma alone from the database. However, the sensitivity of CD-AI for diagnosing glaucoma in eyes with RVO was 0.375, whereas the specificity was 1.0.

### 3.2. Diagnostic Accuracy of the RVO-GLA AI

**Figure 1:**
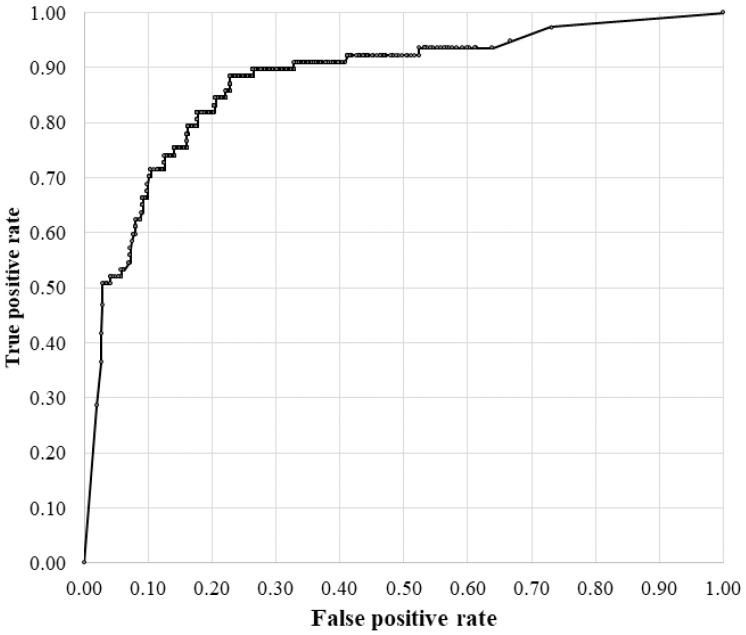
ROC Curve for an RVO-GLA AI for the Diagnosis of Glaucoma.

Compared with CD-AI, the RVO-GLA AI demonstrated improved diagnostic accuracy for glaucoma. For images of eyes with glaucoma, the sensitivity and specificity of the RVO-GLA AI were 0.790 and 0.755, respectively. The receiver operating characteristic (ROC) curve for detecting glaucoma in all tested images is shown in Figure 1, with an area under the curve (AUC) of 0.8755.

### 3.3. Comparison of the RVO-GLA AI with Ophthalmologists

**Figure 2:**
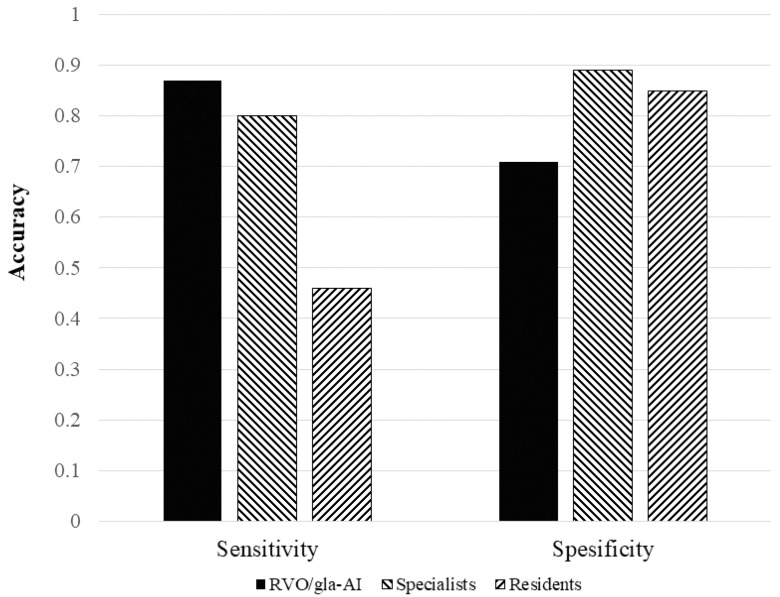
Comparison of the sensitivity and specificity between the RVO-GLA AI and ophthalmologists.

As shown in Figure 2, the RVO-GLA AI achieved a sensitivity and specificity comparable to those of ophthalmology specialists and showed better sensitivity than ophthalmology trainees. The sensitivity and specificity of the RVO-GLA AI were 0.87 (95% CI, 0.63–0.96) and 0.71 (95% CI, 0.57–0.82), respectively. In comparison, specialists showed a sensitivity of 0.80 (95% CI, 0.56–0.93) and a specificity of 0.89 (95% CI, 0.77–0.95), whereas residents showed a sensitivity of 0.46 (95% CI, 0.25–0.69) and a specificity of 0.85 (95% CI, 0.73–0.92). These results indicate that the ability of the RVO-GLA AI to diagnose glaucoma in fundus images of eyes with RVO is similar to that of ophthalmology specialists, while providing higher sensitivity than ophthalmology trainees.

McNemar’s test showed no significant difference in overall diagnostic accuracy between the RVO-GLA AI and specialists (*p* = 0.10), or between the RVO-GLA AI and residents (*p* = 0.65).

## 4. Discussion

Our study demonstrated that fine-tuning an existing AI-based glaucoma diagnostic model by incorporating a limited number of eyes with concomitant RVO improved diagnostic performance in this complex clinical scenario and achieved accuracy comparable to that of ophthalmologists. Many previous studies have focused on differentiating eyes with glaucoma from healthy eyes and have achieved high diagnostic accuracy using fundus color images [3,4,5,6,7,8,9,10]. However, in real-world clinical settings, patients often present with multiple cooccurring fundus diseases. Few studies have addressed the challenge of distinguishing glaucoma from other fundus diseases, highlighting the gap in the applicability of AI algorithms to complex cases.

**Figure 3:**
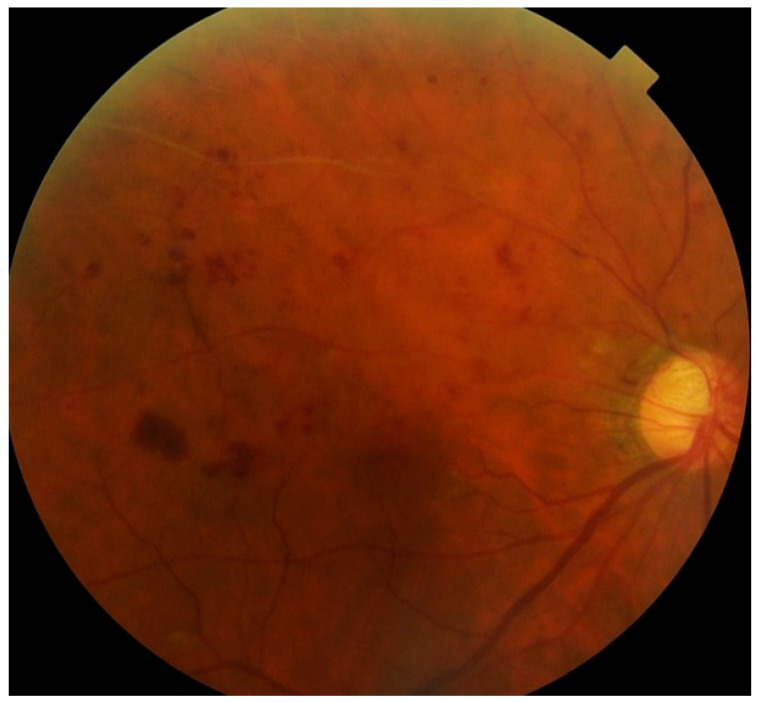
A representative case in which CD-AI was misdiagnosed as RVO only.

This study revealed that the CD-AI algorithm, designed to diagnose single fundus diseases, exhibited poor diagnostic accuracy for glaucoma complicated by RVO. A representative case is shown in Figure 3, where CD-AI misdiagnosed a 78-year-old male patient with glaucoma and branch RVO; this patient was labeled as having RVO with a 99.99% probability of not having glaucoma. Such misdiagnoses can lead to delayed glaucoma detection, as patients often remain asymptomatic until advanced glaucomatous damage occurs.

The prevalence of glaucoma and RVO in the Japanese population over 40 years of age is 5.0% and 2.3%, respectively [11,12,13,14], making their coexistence relatively common. These findings underscore the necessity of algorithms capable of detecting glaucoma in patients with RVO.

In this study, we developed the RVO-GLA AI algorithm, which achieved a diagnostic accuracy equivalent to that of ophthalmology specialists. Using a two-step strategy, the algorithm was trained on a relatively small number of fundus images, including those from patients with combined glaucoma and RVO. By gradually increasing the proportion of combined cases to approximately 1% in the training set, the model’s AUC significantly improved to 0.8755. This approach demonstrates that even a small number of complex cases can enhance algorithm performance, a finding with significant implications for developing AI models for multiple cooccurring diseases.

**Figure 4:**
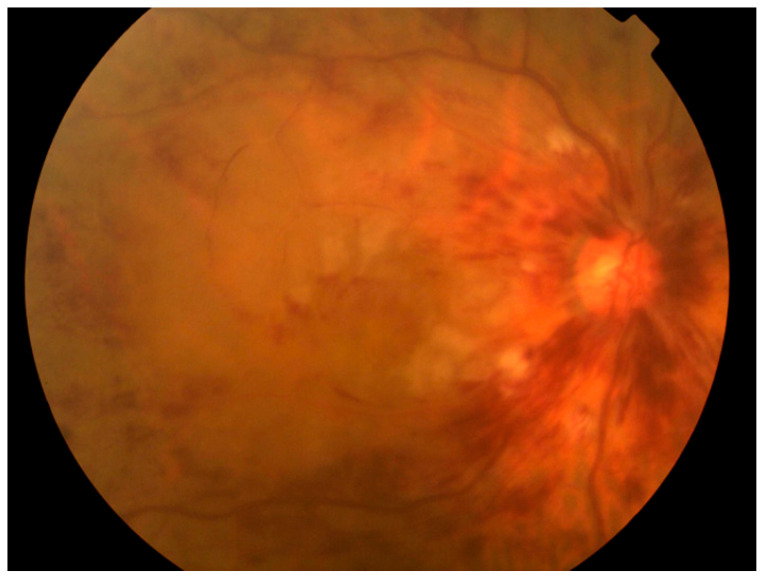
A representative case in which extensive hemorrhaging due to RVO-obscured glaucomatous features.

**Figure 5:**
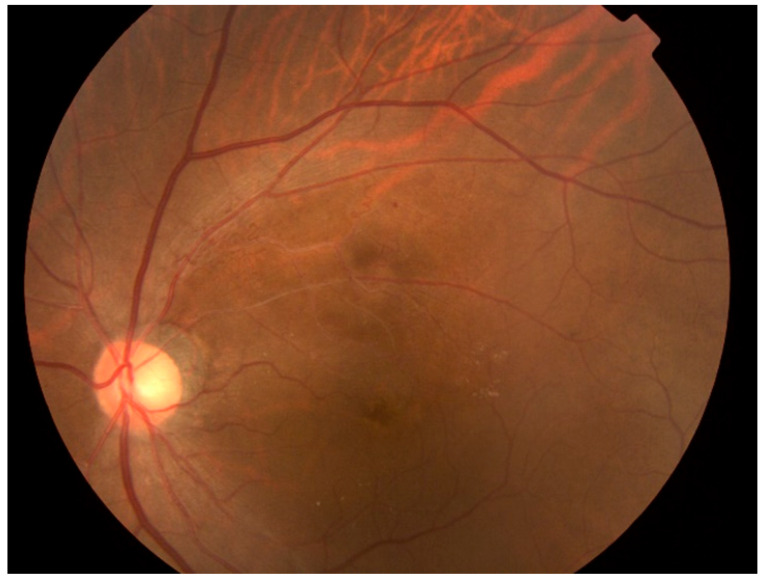
A representative case of chronic RVO that led to a misjudgment of glaucoma.

Despite these advancements, several limitations remain. A representative case misdiagnosed by the RVO-GLA AI is shown in Figure 4, where extensive hemorrhaging due to RVO-obscured glaucomatous features. The misclassification in hemorrhagic RVO cases likely resulted from a scarcity of such samples in the training data, and increasing the representation of these severe presentations may further improve the robustness of the model. Conversely, Figure 5 illustrates a case of misjudgment, where the RVO-GLA AI incorrectly identified glaucoma in a patient with chronic RVO but no glaucomatous cupping or history. These findings emphasize the need for further optimization of the algorithm and the inclusion of diverse and representative datasets.

Additionally, the variability in fundus images due to active or chronic RVO and different glaucoma presentations highlights the necessity of increasing the sample size to confirm real-world utility. Another limitation of this study is the potential bias related to the study population and imaging devices. Since all training and evaluation images were obtained from Japanese patients at a limited number of institutions using similar non-mydriatic fundus cameras, the generalizability of the RVO-GLA AI to other ethnicities and to images acquired with different devices remains uncertain and should be validated in future studies. Furthermore, as diagnostic algorithm performance may vary across racial groups, future studies must assess the generalizability of these findings to other populations.

This study provides a foundation for developing AI models to diagnose complex cases involving multiple fundus diseases. Future research should expand to include other cooccurring conditions, such as diabetic retinopathy with glaucoma (DM-GLA) and age-related macular degeneration with glaucoma (AMD-GLA). Determining the optimal proportion of combined cases in training datasets and validating the algorithm’s performance in diverse populations will be essential for broader clinical applicability.

## 5. Conclusions

This study successfully developed the RVO-GLA AI algorithm, which is capable of diagnosing glaucoma in eyes with RVO with an accuracy comparable to that of ophthalmology specialists. Using a relatively small number of training images, this approach indicated the potential feasibility of AI-based clinical decision support tools capable of identifying coexisting fundus diseases in clinical settings, such as glaucoma in eyes with RVO. The algorithm has the potential to assist in detecting glaucoma in patients with RVO and may serve as a reference for developing diagnostic support systems for other clinically relevant combinations of ocular diseases; however, further validation in larger and more diverse populations is required before broad clinical implementation.

## Data Availability

The data presented in this study are not publicly available due to ethical and privacy restrictions. De-identified data may be made available from the corresponding author upon reasonable request and with approval from the Ethics Committee of the University of Yamanashi.
